# Application of Persimmon Pectin with Promising Emulsification Properties as an Acidified Milk Drinks Stabilizer

**DOI:** 10.3390/foods12102042

**Published:** 2023-05-18

**Authors:** Lanlan Hu, Yangyang Jia, Xiaoxiao Zhang, Yajie Zhang, Meizhu Dang, Chunmei Li

**Affiliations:** 1 College of Food Science and Technology, Huazhong Agricultural University, Wuhan 430070, China; 2 Environment Correlative Food Science, Ministry of Education, Wuhan 430070, China; 3 School of Food Science, Henan Institute of Science and Technology, Xinxiang 453003, China

**Keywords:** persimmon pectin, stability, acidified milk drinks, natural ingredients

## Abstract

The present study aimed to evaluate the capability of persimmon pectin (PP) as a stabilizer for acid milk drinks (AMDs) compared with commercial high-methoxyl pectin (HMP) and sugar beet pectin (SBP). The effectiveness of pectin stabilizers was assessed by analyzing particle size, micromorphology, zeta potential, sedimentation fraction, storage, and physical stability. Results of CLSM images and particle size measurements showed that PP-stabilized AMDs had smaller droplet sizes and more uniform distributions, indicating better stabilization potential compared with the HMP- and SBP-stabilized AMDs. Zeta potential measurements revealed that the addition of PP significantly increased the electrostatic repulsion between particles and prevented aggregation. Moreover, based on the results of Turbiscan and storage stability determination, PP exhibited better physical and storage stability compared with HMP and SBP. The combination of steric repulsion and electrostatic repulsion mechanisms exerted a stabilizing effect on the AMDs prepared from PP. Overall, these findings suggest that PP has promising potential as an AMD stabilizer in the food and beverage industry.

## 1. Introduction

Acidified milk drinks (AMDs) are a category of food systems including drinking yogurt, fruit milk drinks, and soft drinks with milk solids as a minor component [1,2]. AMDs provide a good source of protein, and there is a growing consumer demand for ready-to-drink protein beverages [3]. Over the past few decades, AMDs have become increasingly popular and are now produced on a large scale. The predominant proteins in bovine milk are caseins, which naturally exist in colloidal particles known as casein micelles, comprising approximately 80% of milk proteins [4]. At neutral pH, caseins remain stable within micelles due to the steric repulsion resulting from the extended conformation of K-casein [5]. However, the final pH of AMDs generally falls between 3.6 to 4.6, which causes casein micelles to form into colloidal particles, despite the improved texture [6]. This leads to an industrial defect called “wheying off”, which is primarily caused by the reduction in steric repulsion on the surface of casein micelles during the acidification process [7]. Consumers expect AMDs to have the same visual homogeneity as milk [2,8]. Therefore, stabilizers are required to be added to prevent the flocculation of milk proteins and the resulting macroscopic whey separation. The development of a stable, visually appealing dairy drink with low pH poses a challenge for dairy-based beverage manufacturers. There is considerable interest in formulating food products with natural ingredients to meet the consumer demand for “cleaner labels” and “natural ingredients” due to their perceived healthiness and sustainability [9,10].

The use of anionic hydrocolloids to stabilize AMDs has generated significant interest in the past decade [7,11]. Introducing anionic polysaccharides as stabilizers can mitigate casein aggregation in AMDs, as the anionic polysaccharides could modify the functional behavior of milk proteins and prevent serum separation in acidified milk beverages [8,12]. Pectin, a common anionic polysaccharide, is composed of α-1,4-linked d-galacturonic acid (GalA) chains and neutral sugars, and is typically used as a gelling agent, thickener and emulsifier [13,14]. Presently, commercial pectin is mainly extracted from citrus, sugar beet or apple pomace. Interestingly, pectin exhibits good emulsifying properties in acidic environments, making it possible to stabilize AMDs [15]. The degree of methoxylation (DM) determines whether pectin carries a negative charge at pH scales above 3.5 [16]. At pH < 4.6, the casein micelle surfaces carry a net positive charge [2]. Therefore, at the pH typical of AMDs (approximately 4.0), electrostatic interaction occurs between negatively charged pectin and positively charged casein micelles [17]. However, pectin’s interaction with casein micelle surfaces may vary along the entire chain due to its complex structure [12]. Several factors, such as milk solid content, pectin type, concentration, molecular characteristics (e.g., molecular weight, DM, and charge density), environmental conditions (e.g., pH, temperature, and ionic strength), and processing factors (e.g., homogenization and thermal treatment) may affect the pectin–casein interaction and the resultant stability of AMDs [16]. Compared with citrus pectins, sugar beet pectin (SBP) is not yet widely used in food applications, despite the excellent emulsification performance of SBP. For example, high-methoxyl pectin (HMP) derived from citrus is commonly used to stabilize AMDs and prevent casein flocculation [18], while limited research has been conducted on SBP as a stabilizer for AMDs [19]. HMP has been proven to show better stabilization at higher pH scales (4.0 and 4.2) but is less effective under lower pH values (3.4, 3.6, and 3.8) [20]. In this study, the molecular parameters of the HMP and SBP used were as follows: commercial SBP (Mw: 4.613 × 10^5^ g/mol; protein content: 21.32 ± 1.41 mg/g; DM: 68.29% ± 1.38%) was provided by CP Kelco (Lille Skensved, Denmark), and HMP (Mw: 6.443 × 10^5^ g/mol; protein content: 6.42 ± 0.37 mg/g; DM: 59.38% ± 1.01%) was obtained from Aladdin (Shanghai, China).

Persimmon (*Diospyros kaki*) is considered a functional fruit due to its health-enhancing benefits [21,22]. China is the leading producer of persimmons globally, and our previous studies have shown that persimmon pectin (PP) exhibits promising emulsification properties [23,24]. However, it has not yet been reported whether PP can be used as a stabilizer in AMDs. Given the potential benefits of using a low-cost and health-promoting stabilizer, the main objective of this study was to investigate the effectiveness of PP as an AMDs stabilizer. Based on previous research on the structural characteristics of PP, as well as its emulsification properties, the hypothesis was that PP could be a viable stabilizer for AMDs.

The study aimed to evaluate the capability of PP to stabilize AMDs compared with HMP and SBP. The efficacy of PP as an AMD stabilizer was analyzed by droplet size, micromorphology, zeta potential, sedimentation fraction, storage and physical stability. The results demonstrate the potential of PP as a promising stabilizer for AMDs, suggesting its potential applications in the food and beverage industry.

## 2. Materials and Methods

### 2.1. Materials

Fresh persimmon fruit (*Diospyros kaki*) was obtained from a local market (Baishazhou Market, Wuhan, China). The extraction of pectin from persimmon fruit was performed as described in our previous study [23,24]. Skim milk powder, containing 35.2% protein, 55.2% carbohydrate, 1.2% calcium and maximum 1.5% fat, was obtained from Yili Industrial Group (Inner Mongolia, China). Nile blue dye was procured from Shanghai Yuanye Bio-Technology Co., Ltd. (Shanghai, China). All solutions were prepared using deionized water.

### 2.2. Preparation of AMDs

To assess the effectiveness of pectic polymers in stabilizing AMDs, samples were prepared at three different concentration levels (*w*/*v*): 0.5%, 1.0%, and 2.0%. Skim milk powder (6.67%, *w*/*v*) and pectin samples (0.5–2.0%, *w*/*v*) were separately dissolved in deionized water by stirring in a water bath at 40 °C, and then mixed in a 1:1 ratio. The final volume of the sample was 100 mL, with a protein concentration of 1.0% (*w*/*v*) and pectin concentrations of 0.25% (*w*/*v*), 0.5% (*w*/*v*), and 1.0% (*w*/*v*) in the final AMDs.

To facilitate the stabilization of casein particles by pectins, the mixture was homogenized with an IKA homogenizer (Staufen, Germany) at 8000 rpm for 3 min. The pH value was acidified by stepwise adding citric buffer. The final products were stored at 4 °C.

### 2.3. Particle Size Characterization

Particle size (D_32_ and D_43_) and distribution measurements were performed using a Mastersizer 2000 (Malvern Instruments, Worcestershire, UK). The refractive indices of 1.335 and 1.348 were used for the aqueous phase (i.e., deionized water) and the dispersed particles, as recommended for low-fat dairy products [25]. Samples were introduced into a flow of degassed water until the obscuration was around 10%. All the measurements were conducted under ambient conditions.

### 2.4. Micromorphology Observation

Microstructure of pectin-stabilized AMDs was visualized using a confocal scanning laser microscope (FV3000, Olympus, Japan). Nile blue (0.1% in ethanol) was added to the AMDs to label the proteins, and the samples were shaken for 2 min to ensure complete dyeing. Then, 30 μL of dyed sample was dripped onto a hollow slide and covered with a coverslip hermetically. The sample was observed with 40× magnification objective lens under 633 nm excitation wavelength of Ar laser and bright field in a CLSM system.

### 2.5. Zeta Potential and Sedimentation Measurement

The zeta potential of AMDs was measured using the Zetasizer Nano ZS (Malvern Instruments, Worcestershire, U.K.) at 25 °C after diluting 100-fold in deionized water.

Sedimentation fraction was determined by centrifuging ten grams of AMDs at 2000× *g* for 20 min at 25 °C. After centrifugation, the supernatant was decanted, and the remaining supernatant was drained by inverting the centrifugation tube for 10 min. The sedimentation fraction was calculated by dividing the weight of the residual precipitate by the weight of the sample.

### 2.6. Stability Evaluation of AMDs

Stability is a critical factor not only in determining the shelf life of AMDs but also in evaluating the effectiveness of stabilizers [26]. In this section, the stability of AMDs in terms of both storage and physical stability was evaluated.

#### 2.6.1. Storage Stability Evaluation of AMDs

In this study, fresh AMDs were transferred into sample bottles and stored in the dark at 4 °C for 7 d. The particle size (D_32_ and D_43_) and zeta potential of the AMDs were determined.

#### 2.6.2. Physical Stability Evaluation of AMDs

To assess the physical stability of the AMDs in real-time, a Turbiscan Tower (Formulation, Toulouse, France) was employed, which uses near-infrared light with a wavelength of 850 nm. Test tubes were filled up to 45 mm with the freshly prepared AMDs. Then, they were scanned 96 times with an interval of 10 min between each scan. The stability of AMDs was evaluated based on changes in transmission, back-scattering profile (BS), Turbiscan stability index (TSI), and separation layer thickness.

### 2.7. Data Analysis

AMDs without added pectin stabilizer were considered as controls. All treatments were conducted in triplicate, and analysis of variance (ANOVA) by SPSS 19.0 software (SPSS Inc., Chicago, IL, USA, version 19.0) at a significance level of 0.05 was performed. All figures were plotted using Origin 2018 software.

## 3. Results

### 3.1. PP-Stabilzied AMDs Exhibited Smaller Particle Size and Narrower Size Distribution Compared with Commercial Pectins

According to Stokes’ law, the velocity of droplet motion is proportional to the square of its radius. Therefore, the stability of AMDs might be related to their droplet size [26]. In unstable AMDs, casein micelles tend to flocculate and aggregate into large particles, leading to macroscopic phase separation with whey on the upper layer and sediment on the bottom. Thus, the effectiveness of pectin as a stabilizer for AMDs can be assessed by measuring particle size (D_32_ and D_43_) and distributions, with smaller D_32_ and D_43_ values and narrower particle size distribution indicating better stabilization capacity of AMDs by pectins [19].

**Figure 1:**
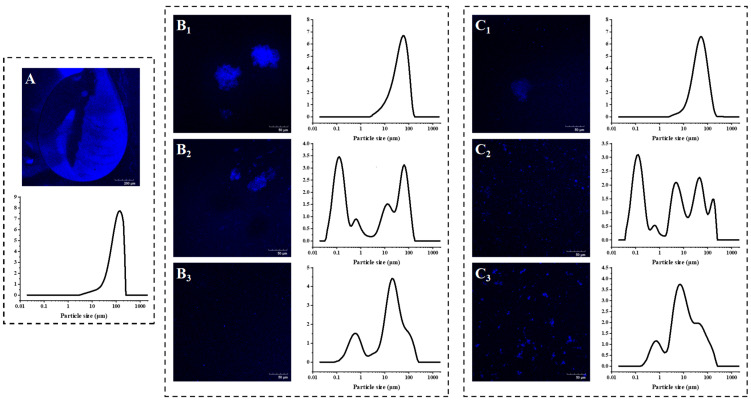
Confocal laser scanning microscopic images combined with particle size distribution curves of pectin-stabilized AMDs: (**A**) Control; (**B**) HMP; (**C**) PP (Subindex 1: 0.25%, *w*/*v*; Subindex 2: 0.5%, *w*/*v*; Subindex 3: 1.0%, *w*/*v*). Note: Control—AMD without added pectins; PP—persimmon pectin; HMP—high-methoxyl pectin from citrus.

As shown in Table 1, except for SBP, the D_43_ value of AMDs with 0.25–1.0% of pectins (*w*/*v*) as stabilizers was much smaller than that of AMDs without stabilizers (113.20 ± 1.90 μm). Additionally, the D_32_ and D_43_ values of PP (0.25%, 0.5%, and 1.0%, *w*/*v*) stabilized AMDs were smaller than those of commercial pectins (HMP and SBP), suggesting that PP has higher stabilizing capability for AMDs. The droplet size distribution curves combining CLSM images were further evaluated to illustrate the droplet morphology of pectin-stabilized AMDs. A stable AMDs system typically displays uniform droplet size distributions with smaller particles, while the emergence of large aggregates with tens to hundreds of micrometers indicates an unstable system [7,18]. As shown in Figure 1A, an initial monomodal distribution with particle sizes of 2–200 μm was observed in the AMDs without added pectins (i.e., control), and large aggregated clusters of casein were observed in CLSM microscopy. The volume distribution shifted from monomodal to multimodal peaks after adding HMP and PP, with new peaks in the small particle region, indicating that the addition of PP and HMP augmented the number of small particles. Notably, Figure 1C shows that a more uniform particle size distribution and smaller particle size of AMDs was observed after PP addition, suggesting that PP significantly improved the stability of AMDs.

In detail, as the PP concentration increased from 0.25% to 0.5% (*w*/*v*), the D_43_ value of AMDs significantly decreased from 55.52 ± 5.60 to 28.79 ± 4.62 μm, and the large aggregates in the microscopic images were declustered, indicating that 0.5% (*w*/*v*) PP effectively inhibited the aggregation of casein micelles. Decreased particle size significantly reduced the precipitation of milk protein dispersions stabilized by pectins in AMDs. However, as the PP concentration increased further to 1.0% (*w*/*v*), the D_43_ value of AMDs changed insignificantly, and the D_32_ value increased instead. In addition, partial aggregation was observed in the microscopic images (Figure 1C), probably due to the occurrence of depletion flocculation induced by 1.0% (*w*/*v*) PP. Therefore, 0.5% (*w*/*v*) PP was sufficient to stabilize AMDs. Similarly, as the HMP concentration increased from 0.25% to 0.5% (*w*/*v*), the D_43_ value of AMDs significantly decreased from 62.61 ± 13.04 to 33.15 ± 6.76 μm, and the large aggregates in the microscopic images were partially declustered (Figure 1B). In addition, as the HMP concentration increased to 1.0% (*w*/*v*), the D_43_ decreased further, and complete declustering of the aggregates was observed in the microscopic images (Figure 1C). Therefore, the distribution of AMDs stabilized by HMP shifted from aggregate to uniform as the concentration increased, and a 1.0% (*w*/*v*) of HMP was required to stabilize AMDs. Interestingly, Wei et al. [26] indicated that the optimal concentration of HMP was 1% for stabilizing pea protein dispersions. Moreover, the large D_43_ value (0.25%, *w*/*v*: 184.49 ± 5.53 μm; 0.5%, *w*/*v*: 135.86 ± 2.01 μm; and 1.0%, *w*/*v*: 111.94 ± 47.90 μm) and broad size distribution of SBP-prepared AMDs were observed due to the formation of large aggregates. Therefore, the capability of SBP to stabilize AMDs was weak.

The analysis of droplet size characteristics and CLSM observations supported that PP has superior stabilization properties for AMDs compared with commercial pectins (HMP and SBP), and 0.5% (*w*/*v*) concentration of PP was identified as the baseline for avoiding protein precipitation under acidic conditions.

### 3.2. PP-Stabilized AMDs Exhibited Strong Electrostatic Repulsion Than SBP and Comparable to HMP

Zeta potential is commonly used to characterize the surface charge of colloidal casein and to assess the magnitude of electrical charge on particles [6]. Table 2 shows the zeta potentials of AMDs prepared with different concentrations of PP, HMP and SBP. Notably, as they were below the isoelectric point of casein, AMDs prepared without stabilizers (i.e., control) were positively charged (+1.70 ± 0.54 mV). Similarly, the zeta potential of AMDs was around +1 mV without any hydrocolloid [27]. In contrast, the zeta potential of AMDs stabilized with the three pectin stabilizers shifted towards negative values. It has been suggested that the shift was because of the adsorption of anionic pectin molecules onto the positively charged proteins. The same trend was found for AMDs stabilized by soluble soybean polysaccharides and potato pectin [3,17]. Moreover, the zeta potential (absolute) of AMDs increased with the augmented pectin concentrations. Similarly, the zeta potential (absolute) of AMDs stabilized by HMP, LMP, and potato pulp pectin showed a decreasing trend with the increasing pectin concentration [17].

Generally, pectin-coated casein particles with larger zeta potential (absolute) typically indicate greater stability due to increased electrostatic repulsion [26]. In this study, the zeta potential values of AMDs prepared with PP (ranging from −19.86 ± 1.18 to −26.80 ± 0.94 mV) were generally higher than those of SBP (ranging from −5.14 ± 1.15 to −22.71 ± 2.81 mV) but comparable to HMP (ranging from −18.52 ± 2.61 to −28.63 ± 0.43 mV). These results suggest that the electrostatic repulsion of casein colloidal covered with PP was stronger than SBP and comparable to HMP. Previous studies have shown that electrostatic repulsion was the major force contributing to the stability of AMDs prepared with HMP [2,18]. Thus, the strong electrostatic repulsion between casein–PP likely played a crucial role in preventing the phase separation of casein particles in AMDs.

### 3.3. PP Exhibited Lower Sedimentation Rate Compared with Commercial Pectins

The ability of different pectins to stabilize AMDs was further investigated through sedimentation measurements. The percentage of precipitation in AMDs is a direct and fundamental index for measuring stability, with lower sedimentation levels indicating better stability of milk proteins under acidic conditions.

As shown in Table 2, AMDs without stabilizers sedimented immediately after acidification, with a sedimentation rate of 34.33% ± 0.01%. However, AMDs stabilized with 0.5% (*w*/*v*) of PP and HMP had lower sedimentation rates, at 19.00% ± 4.58% and 20.33 ± 1.53%. The amount of sedimentation in AMDs decreased significantly with increasing pectin concentration (0.25% to 1.0%, *w*/*v*). Furthermore, PP was found to be more effective in protein stabilization under acidic conditions than commercial pectins (HMP and SBP), as evidenced by the lower percentage of sedimentation in AMDs stabilized by PP compared with those stabilized by HMP or SBP at the same concentration.

In summary, the results suggest that PP could effectively stabilize milk proteins under acidic conditions in AMDs.

### 3.4. PP Exhibited Higher AMDs Storage Stability Compared with Commercial Pectins

Stability is a crucial factor in determining the shelf-life of commercial AMDs. In this study, the storage stability of AMDs with added pectin was assessed by storing the samples in glass tubes for 7 d at 4 °C. The changes in particle size, zeta potential, and amount of whey separation were evaluated.

As shown in Table 3 and Table 4, AMDs prepared with 0.5% and 1.0% (*w*/*v*) PP exhibited decreased D_43_ and increased D_32_ values after 7 d storage, while AMDs stabilized with 0.5% and 1.0% (*w*/*v*) HMP showed increased D_43_ and D_32_ values. Additionally, the D_43_ and D_32_ values of SBP-prepared AMDs significantly increased.

As shown in Table 5, The zeta potential (absolute) of 0.25% (*w*/*v*) pectin-stabilized AMDs significantly reduced, indicating a decrease in electrostatic repulsion between droplets. However, the zeta potential (absolute) of AMDs prepared with 0.5% and 1.0% (*w*/*v*) PP and HMP changed insignificantly after 7 d storage, maintaining a strong electrostatic repulsion. In contrast, the zeta potential (absolute) of AMDs prepared with SBP was significantly reduced after 7 d storage, especially those of AMDs prepared with 0.25% and 0.5% (*w*/*v*) SBP, which decreased substantially and were close to zero.

**Figure 2:**
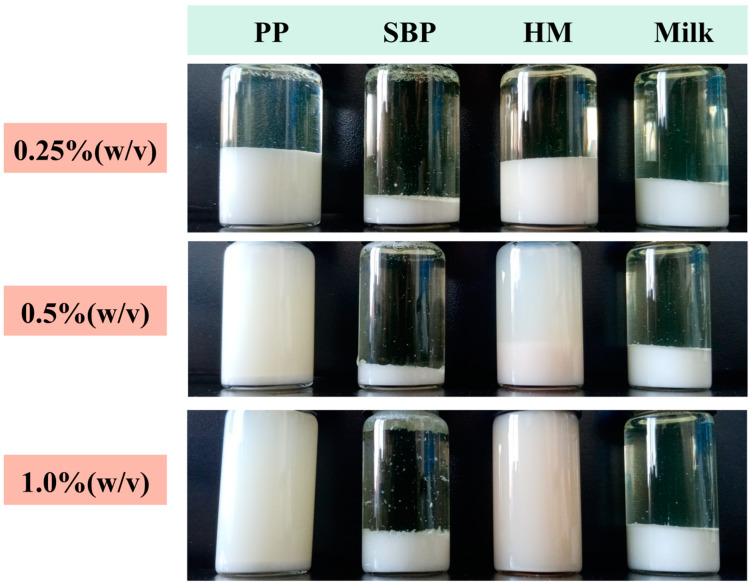
Photograph of pectin-stabilized AMDs after 7 d storage; Note: PP—persimmon pectin; SBP—sugar beet pectin; HMP—high-methoxyl pectin from citrus.

As shown in Figure 2, AMDs without pectins (i.e., control) exhibited complete creaming separation, and the whey layer was not visually turbid. When pectin was added to AMDs, the amount of creaming decreased with increasing pectin concentration. Notably, significant whey separation was observed for all concentrations of SBP (0.25–1.0%, *w*/*v*). Significant separation was also observed for AMDs prepared with 0.25% (*w*/*v*) PP and HMP after 7 d storage, indicating that the amount of pectin was insufficient to saturate the positively charged surface of the micelles, leading to the occurrence of bridging flocculation. Sejersen et al. [28] indicated that 0.3% pectin was insufficient to stabilize AMDs. Similarly, Kermani et al. [12] reported that 0.25% (*w*/*v*) mango peel pectin and apple pectin were insufficient to stabilize AMDs, presumably due to the low pectin concentration not being sufficient to stabilize casein aggregates. At the same pectin concentration (0.25% and 0.5%, *w*/*v*), PP exhibited higher AMD-stability potential than HMP, although it did not completely prevent phase separation at 0.25% (*w*/*v*). Furthermore, at 1.0% (*w*/*v*) concentration, PP and HMP significantly inhibited whey separation and presented a milk-like appearance.

### 3.5. PP Exhibited Higher AMDs Stability Potential Based on Multi-Light Scattering Compared with Commercial Pectins

In addition to the natural phase separation during storage, the physical stability of AMDs can be precisely monitored using a Turbiscan Tower, which employs multi-light scattering (transmission and backscattering) to record the optical properties of samples in real-time. Notably, data are selectively presented every 4 h.

**Figure 3:**
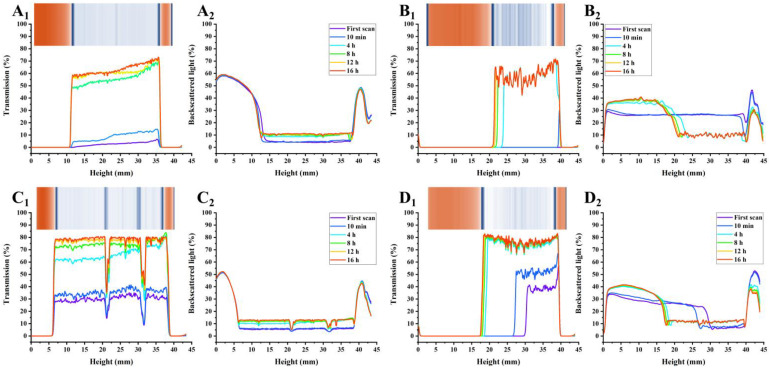
Transmission (Subindex 1) and back-scattering (Subindex 2) profiles of AMDs stabilized by pectins (0.25%, *w*/*v*) during 16 h detection, and simulation diagrams (insets) after 16 h detection ((**A**): Control; (**B**): PP; (**C**): SBP; (**D**): HMP); Note: Control—AMD without added pectins; PP—persimmon pectin; SBP—sugar beet pectin; HMP—high-methoxyl pectin from citrus.

**Figure 4:**
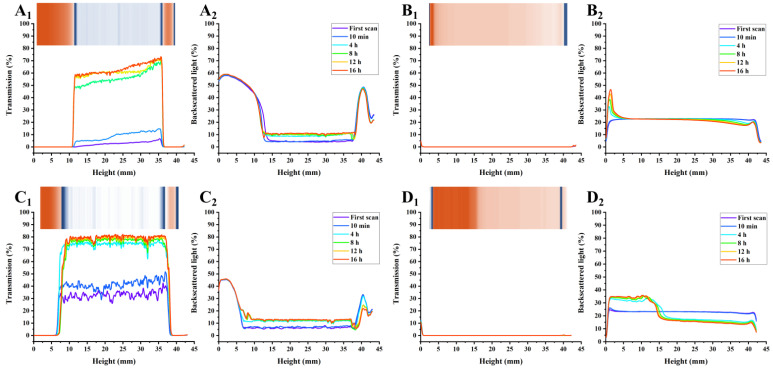
Transmission (Subindex 1) and back-scattering (Subindex 2) profiles of AMDs stabilized by pectins (0.5%, *w*/*v*) after 16 h detection, and simulation diagrams (insets) after 16 h detection ((**A**): Control; (**B**): PP; (**C**): SBP; (**D**): HMP); Note: Control—AMD without added pectins; PP—persimmon pectin; SBP—sugar beet pectin; HMP—high-methoxyl pectin from citrus.

**Figure 5:**
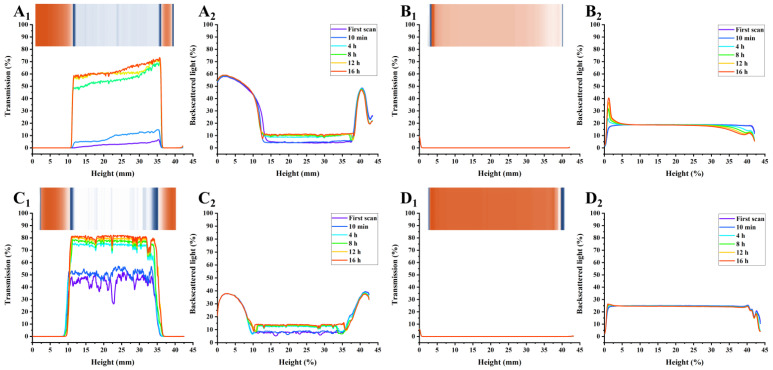
Transmission (Subindex 1) and back-scattering (Subindex 2) profiles of AMDs stabilized by pectins (1.0%, *w*/*v*) during 16 h detection, and simulation diagrams (insets) after 16 h detection ((**A**): Control; (**B**): PP; (**C**): SBP; (**D**): HMP); Note: Control—AMD without added pectins; PP—persimmon pectin; SBP—sugar beet pectin; HMP—high-methoxyl pectin from citrus.

**Figure 6:**
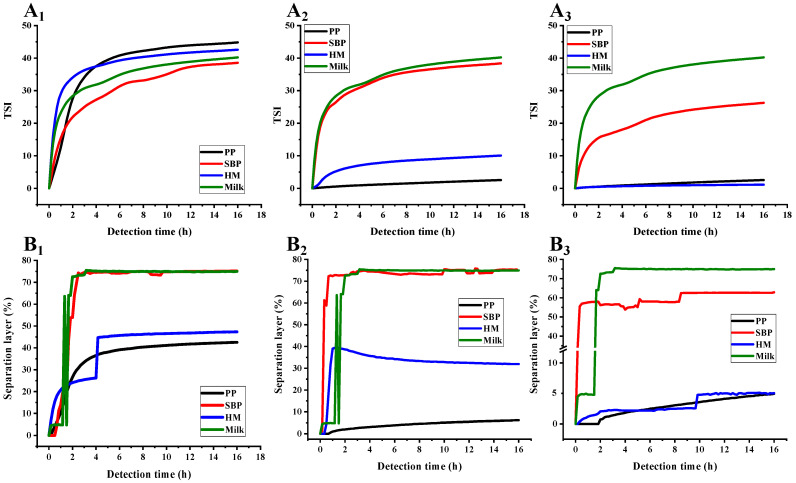
Destabilization kinetic of AMDs prepared by pectins during detection. Turbiscan stability index (TSI) of AMDs for 16 h (**A**), and separation layer (**B**) of AMDs for 16 h (Subindex 1: 0.25%, *w*/*v*; Subindex 2: 0.5%, *w*/*v*; Subindex 3: 1.0%, *w*/*v*); Note: PP—persimmon pectin; SBP—sugar beet pectin; HMP—high-methoxyl pectin from citrus.

As shown in Figure 3, Figure 4 and Figure 5, the transmission and backscattering graphs for PP-stabilized AMDs exhibited less variation than the other two commercial pectin-prepared AMDs, suggesting the minimal changes in the internal structure of the PP-stabilized AMDs during detection. Turbiscan Stability Index (TSI) represents the comprehensive stability of detected samples, with higher TSI levels indicating less stability of AMDs [29]. Destabilization kinetics were constructed by representing the TSI evolution over 16 h. As shown in Figure 6A, the TSI scale increased with detection, suggesting that the stability of samples decreased during measurements. Except for the 0.25% (*w*/*v*) concentration, the TSI of pectin-stabilized AMDs followed the order of SBP > HMP > PP, indicating the highest AMDs-stability capacity of PP and the least of commercial SBP.

The separation phenomenon was quantitatively monitored by recording the separation thickness of AMDs in real-time with the Turbiscan instrument. As charted in Figure 6B, the highest serum separation (74.98%) occurred in AMDs without pectin stabilizers (i.e., control). Similarly, Sun et al. [17] found that the highest serum separation rate (62.5%) occurred in AMDs without any pectin stabilizer. In addition, the amount of serum separation decreased with increasing pectin concentrations from 0.25% to 1.0% (*w*/*v*). Taking the pectin concentration of 0.5% (*w*/*v*) as an example, SBP exhibited the highest separation layer (75.44%), followed by HMP (31.93%) and PP (6.16%). Thus, PP resulted in a much lower amount of serum separation than SBP and HMP. Sun et al. [17] confirmed that potato pulp pectin was a reliable stabilizer for AMDs. However, when using a defined study concentration (0.5%), the serum separation of AMDs stabilized by potato pulp pectin (14.65%) was observed to be higher than that of HMP (9.09%). Taking into account previous studies and the serum separation evaluation, it can be concluded that PP holds promising potential as a stabilizer for AMDs.

The insets in Figure 3, Figure 4 and Figure 5 show the simulated diagrams of AMDs after 16 h detection. Without pectin stabilizers, significant serum separation occurred in AMDs, whereas the presence of pectins significantly improved the stability of AMDs. Notably, the simulation diagrams showed a significant decrease in serum separation when the concentration of HMP and PP increased from 0.25% (*w*/*v*) to 0.5% (*w*/*v*). No serum separation was observed in the 0.5% (*w*/*v*) and 1.0% (*w*/*v*) PP-stabilized AMDs, while partial creaming was observed in the 0.5% (*w*/*v*) HMP-stabilized AMDs, which disappeared at the 1.0% (*w*/*v*) concentration.

Overall, in comparison with commercial pectins, PP-stabilized AMDs exhibited smaller transmission and backscattering variation, TSI scale, and separation layer, indicating promising AMD-stability potential. Importantly, PP was able to stabilize AMDs at lower concentrations than HMP, which was widely known for its excellent AMD-stabilizing capability, suggesting a significant advantage of PP in stabilizing AMDs.

### 3.6. Potential Mechanism of PP as a Stabilizer for AMDs

The stability of AMDs depends on several factors, such as pectin concentration, pectin type, DM, casein concentration and pH scale. Pectin concentration is a crucial factor that affects the physical stability of AMDs. A critical pectin content is required to stabilize AMD systems with defined pH and milk solids content. It should be noted that adding too little or too much pectin might lead to two common instabilities, namely, bridging flocculation and depletion flocculation, which can result in AMDs’ destabilization.

The mechanism by which HMP stabilizes AMDs has been widely investigated. Studies have reported that HMP adsorbs onto casein micelles via electrostatic interaction during acidification. In addition, the adsorbed HMP could prevent the aggregation and sedimentation of casein micelles through steric stabilization [20]. Jensen et al. [18] proposed a stabilization mechanism resembling the combination of steric stabilization and secondary adsorption. Kermani et al. [12] suggested that neutral sugars (NS; rhamnose + arabinose + galactose) of pectins could assist in stabilizing AMDs by preventing casein aggregation via steric hindrance. The higher NS content of PP (13.02%, *w*/*w*) compared with HMP (8.45%, *w*/*w*) might contribute to stabilizing AMDs. In acidic milk drinks, pectin with higher DM was more effective as a stabilizer [20]. In this study, the DM of PP (71.93% ± 1.23%) was higher than HMP (59.38% ± 1.01%), which may contribute to the stabilization of AMDs.

**Figure 7:**
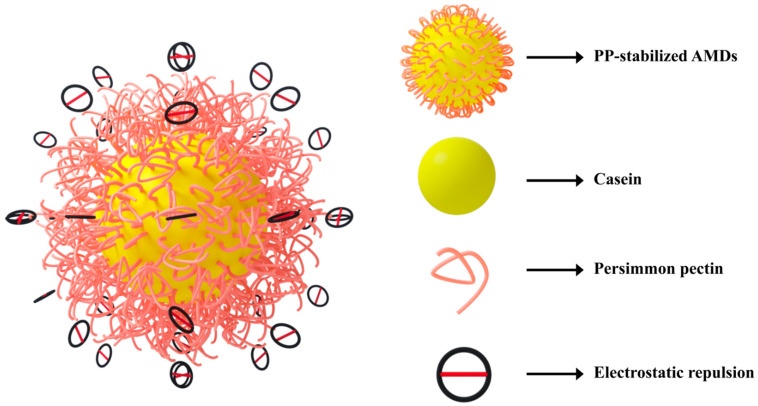
The potential mechanism for the stabilization of AMDs by persimmon pectin.

Our previous study found that the excellent emulsification properties of PP were mainly attributed to strong electrostatic repulsion and steric hindrance. Based on this finding, we hypothesized that in the preparation of AMDs, the negatively charged carboxyl groups in GalA residues of PP bind to positively charged casein micelles, while the non-adsorbed part of the pectin chain provides steric hindrance against casein aggregation. Figure 7 shows a potential mechanism for the stabilization of AMDs by PP that may be attributed to two factors: (1) the greater electrostatic repulsion of the pectin chains, and (2) the thicker layers (possibly multilayers) formed on the particle surface may cause steric repulsion and thus contribute to the stabilization.

## 4. Conclusions

This study investigated the potential of PP as a stabilizer for AMDs and compared its performance with commercial pectins: HMP and SBP. The results showed that AMDs stabilized by PP displayed a more uniform particle size distribution with smaller particle sizes, indicating superior stabilization capacity compared with HMP and SBP. Zeta potential results suggested that PP imparted a negative charge to the casein micelles, contributing to the stability of AMDs. The micromorphology observations further confirmed that PP effectively inhibited the aggregation of casein micelles, making it a promising stabilizer for AMDs. Additionally, the physical and storage stability of AMDs stabilized by PP were improved. The stabilization mechanism of PP-stabilized AMDs involved both steric repulsion and electrostatic repulsion. In conclusion, our findings demonstrated that PP exhibited great potential as a stabilizer for AMDs in the food and beverage industry, providing valuable insights for the development of stable and high-quality AMDs. The spatial conformation of pectins and the performance of AMDs differed greatly depending on the pH. Therefore, further studies are required to elucidate the relationship between pH and AMDs stabilized by pectins, to foster a better understanding and utilization.

## Figures and Tables

**Table 1:** The particle size (D_32_ and D_43_) of pectin-stabilized AMDs.

	Control	PP	SBP	HMP		Control	PP	SBP	HMP
Concentrations	D_32_ (μm)				Concentrations	D_43_ (μm)			
0.25% (*w*/*v*)	64.41 ± 1.66	32.81 ± 2.83↓ ^aA^	81.77 ± 1.93↑ ^bA^	29.51 ± 1.22↓ ^cA^	0.25% (*w*/*v*)	113.20 ± 1.90	55.52 ± 5.60↓ ^aA^	184.49 ± 5.53↑ ^bA^	62.61 ± 13.04↓ ^aA^
0.5% (*w*/*v*)		0.28 ± 0.10↓ ^aB^	84.35 ± 2.62↑ ^bB^	0.35 ± 0.11↓ ^aB^	0.5% (*w*/*v*)		28.79 ± 4.62↓ ^aB^	135.86 ± 2.01↑ ^bB^	33.15 ± 6.76↓ ^aB^
1.0% (*w*/*v*)		3.97 ± 1.02↓ ^aC^	83.47 ± 1.40↑ ^bAB^	1.97 ± 0.76↓ ^cC^	1.0% (*w*/*v*)		27.05 ± 5.43↓ ^aB^	111.94 ± 47.90↑ ^bB^	29.06 ± 5.64↓ ^aB^

Note: Control—AMD without added pectins; PP—persimmon pectin; SBP—sugar beet pectin; HMP—high-methoxyl pectin from citrus. Different lowercase letters in the same row indicate significant differences (*p* < 0.05); different uppercase letters in the same column indicate significant differences (*p* < 0.05). **↓** indicates that the index decreased compared with the control; **↑** indicates that the index increased compared with the control.

**Table 2:** The zeta potential and sedimentation rate of pectin-stabilized AMDs.

	Control	PP	SBP	HMP	Control	PP	SBP	HMP
Concentrations	Zeta Potential (mV)				Sedimentation Rate (%)			
0.25% (*w*/*v*)	1.70 ± 0.54	−19.86 ± 1.18↓ ^aA^	−5.14 ± 1.15↓ ^bA^	−18.52 ± 2.61↓ ^aA^	34.03 ± 0.01	30.78 ± 2.54↓ ^aA^	37.20 ± 1.48↑ ^bA^	28.84 ± 3.39↓ ^aA^
0.5% (*w*/*v*)		−25.11 ± 0.88↓ ^aB^	−5.98 ± 1.36↓ ^bA^	−24.74 ± 1.26↓ ^aB^		19.00 ± 4.58↓ ^aB^	35.00 ± 1.00↑ ^bAB^	20.33 ± 1.53↓ ^aB^
1.0% (*w*/*v*)		−26.80 ± 0.94↓ ^aB^	−22.71 ± 2.81↓ ^bB^	−28.63 ± 0.43↓ ^cC^		16.74 ± 2.65↓ ^aB^	31.75 ± 2.08↓ ^bB^	17.54 ± 1.65↓ ^aB^

Note: Control—AMD without added pectins; PP—persimmon pectin; SBP—sugar beet pectin; HMP—high-methoxyl pectin from citrus. Different lowercase letters in the same row indicate significant differences (*p* < 0.05); different uppercase letters in the same column indicate significant differences (*p* < 0.05). **↓** indicates that the index decreased compared with the control; **↑** indicates that the index increased compared with the control.

**Table 3:** The D_32_ value of pectin-stabilized AMDs before and after storage.

Concentrations	PP (Fresh)	PP (Storage)	SBP (Fresh)	SBP (Storage)	HMP (Fresh)	HMP (Storage)
0.25% (*w*/*v*)	32.81 ± 2.83	37.41 ± 4.96 ^a^	81.77 ± 1.93	85.40 ± 8.93 ^b^	29.51 ± 1.22	30.20 ± 1.60 ^a^
0.5% (*w*/*v*)	0.28 ± 0.10	6.74 ± 0.87 *^a^	84.35 ± 2.62	123.38 ± 2.10 *^b^	0.35 ± 0.11	13.47 ± 0.50 *^c^
1.0% (*w*/*v*)	3.97 ± 1.02	8.07 ± 0.33 *^a^	83.47 ± 1.40	226.79 ± 12.64 *^b^	1.97 ± 0.76	11.67 ± 1.03 *^a^

Note: Control—AMD without added pectins; PP—persimmon pectin; SBP—sugar beet pectin; HMP—high-methoxyl pectin from citrus. * indicates the index of samples after storage significantly changed compared with the fresh samples. Different lowercase letters in the same row indicate significant differences (*p* < 0.05) in the index of pectin-stabilized AMDs after storage.

**Table 4:** The D_43_ value of pectin-stabilized AMDs before and after storage.

Concentrations	PP (Fresh)	PP (Storage)	SBP (Fresh)	SBP (Storage)	HMP (Fresh)	HMP (Storage)
0.25% (*w*/*v*)	55.52 ± 5.60	69.25 ± 4.03 *^a^	184.49 ± 5.53	248.99 ± 68.30 *^b^	62.61 ± 13.04	54.48 ± 2.91 ^a^
0.5% (*w*/*v*)	28.79 ± 4.62	21.84 ± 6.26 ^a^	135.86 ± 2.01	300.10 ± 20.31 *^b^	33.15 ± 6.76	34.85 ± 1.38 ^a^
1.0% (*w*/*v*)	27.05 ± 5.43	14.77 ± 2.81 *^a^	111.94 ± 47.90	551.74 ± 34.44 *^b^	29.06 ± 5.64	38.23 ± 3.96 *^c^

Note: Control—AMD without added pectins; PP—persimmon pectin; SBP—sugar beet pectin; HMP—high-methoxyl pectin from citrus. * indicates the index of samples after storage significantly changed compared with the fresh samples. Different lowercase letters in the same row indicate significant differences (*p* < 0.05) in the index of pectin-stabilized AMDs after storage.

**Table 5:** The zeta potential of pectin-stabilized AMDs before and after storage.

Concentrations	PP (Fresh)	PP (Storage)	SBP (Fresh)	SBP (Storage)	HMP (Fresh)	HMP (Storage)
0.25% (*w*/*v*)	−19.86 ± 1.18	−13.77 ± 3.33 ^a^	−5.14 ± 1.15	−1.97 ± 0.44 *^b^	−18.52 ± 2.61	−10.80 ± 3.31 *^a^
0.5% (*w*/*v*)	−25.11 ± 0.88	−24.94 ± 0.30 ^a^	−5.98 ± 1.36	−1.81 ± 0.70 *^b^	−25.98 ± 1.36	−25.12 ± 1.22 ^a^
1.0% (*w*/*v*)	−26.80 ± 0.94	−25.92 ± 0.89 ^a^	−22.71 ± 2.81	−18.20 ± 0.89 *^b^	−28.63 ± 0.43	−26.79 ± 0.81 *^a^

Note: Control—AMD without added pectins; PP—persimmon pectin; SBP—sugar beet pectin; HMP—high-methoxyl pectin from citrus. * indicates the index of samples after storage significantly changed compared with the fresh samples. Different lowercase letters in the same row indicate significant differences (*p* < 0.05) in the index of pectin-stabilized AMDs after storage.

## Data Availability

The data presented in this study are available on request from the corresponding author.
